# Predictors of Nonadherence to Medications among Hypertensive Patients in Ghana: An Application of the Health Belief Model

**DOI:** 10.1155/2022/1418149

**Published:** 2022-08-24

**Authors:** Fidelis Atibila, Emmanuel Timmy Donkoh, Rob Ruiter, Gerjo Kok, Gill Ten Hoor

**Affiliations:** ^1^Valley View University, Box 183, Techiman, Bono Region, Ghana; ^2^Department of Works and Social Psychology Maastricht University, 616, 6200 MD Maastricht, Maastricht, Netherlands; ^3^Department of Medical Laboratory Science, Box 214, University of Energy and Natural Resources, UENR, Sunyani, Ghana

## Abstract

**Introduction:**

Hypertension (HPT) is recognized as a significant public health problem worldwide from a health and economic perspective. This study determined predictors of nonadherence to HPT medications in Ghana using the health belief model.

**Methods:**

A cross-sectional descriptive survey employing a quantitative approach was conducted among HPT patients who routinely attend clinics at selected hospitals in the Brong Ahafo region of Ghana. Respondents (*n* = 399) were recruited using a multistage sampling technique.

**Results:**

The prevalence of nonadherence was 63.7% (*n* = 254). Nonadherence to hypertension medication was associated with lower education status (*p*=0.009). In logistic regression analysis, patients with high “perceived susceptibility” and “perceived severity” were more likely to forfeit their HPT medication schedules, while patients with high “perceived barriers” and “cues to action” were less likely to skip their medication.

**Conclusion:**

The present study suggests a plausible path to improving medication adherence in this population. Given the high prevalence of nonadherence, policymakers need to urgently design tailor-made health promotion interventions to ensure optimal health outcomes.

## 1. Introduction

Hypertension (HPT) remains an overwhelming global health concern [1, 2]. Despite the implementation of national and global policy agenda to deal with this challenge [2], HPT control remains elusive, resulting in many instances of cardiovascular complications and deaths. According to the World Health Organization (WHO), medication adherence is defined as the extent to which an individual executes lifestyle changes and takes medications as recommended by healthcare professionals [3]. Notwithstanding an increase in HPT awareness globally, adherence is still a lingering problem for patients taking HPT medications [4].

Research carried out in Ethiopia and the Democratic Republic of Congo (DRC) revealed that 46.6% and 15.4% of HPT patients, respectively, had their blood pressure poorly controlled because of nonadherence to HPT medications [5]. This phenomenon of low adherence to HPT medications in lower- and middle-income countries puts a great burden on the healthcare system [6].

The prevalence of HPT in Ghana has gradually and progressively increased over the past two decades [7]. The current prevalence of HPT in Ghana is estimated to be between 30.1% and 40.0%, with an advance in age, excessive alcohol consumption, smoking, and physical inactivity considered as the major health risk factors [8–10]. Studies conducted in deprived areas in Accra reported a 28.3% prevalence of HPT. However, only 7.4% of the study participants were aware of their status; just 4% were on HPT medications; and only a meagre 3.5% had their blood pressure (BP) well managed [11]. A study conducted in the Brong Ahafo region of Ghana among persons taking prescribed HPT medication demonstrated that more than a half of respondents did not comply with their drug schedules [12].

In addition, studies have shown that poor adherence to HPT medications usually leads to increased cardiovascular risk and chronic kidney disease (CKD) [1, 13]. Current estimates reveal that in Ghana, about 31% of the adult population have HPT, presenting a high cost burden to the healthcare system, patients, and their dependents and are at a significant risk of cardiovascular morbidity and mortality as well [1, 14]. Furthermore, nearly half of persons identified with HPT may have evidence of target-organ damage in Ghana due to delay in detection and poor compliance to therapy [15, 16].

Several studies have been conducted to determine the factors contributing to nonadherence to medication. However, only a handful have taken advantage of the insights from the Health Belief Model (HBM) [12, 17, 18]. A better understanding of the HBM and behaviours may be an important reference point for improving the clinical management of HPT and an important gain in controlling the surge in cardiovascular diseases. The HBM is one of the theories used by social psychologists to describe social behaviour as well as health events [19]. It was subsequently expanded to include a variety of health behaviours such as diet, smoking, physical activities, alcohol consumption, and obstetric outcomes [20]. The model contains several constructs that predict why people take actions to control their illness: these are perceived susceptibility, perceived severity, perceived benefits, perceived barriers, and cues to actions. It has the advantage of encouraging people diagnosed with HPT to place a premium on the gains from compliance and also critically examine the potential of developing HPT complications before making a decision [21]. However, there is limited evidence on the application of the HBM in developing countries such as Ghana. It is therefore imperative to determine patients' nonadherence to HPT medication using the HBM to help inform policy formulation and advocacy in the middle belt of Ghana.

## 2. Materials and Methods

### 2.1. Study Design

A cross-sectional descriptive survey employing a quantitative approach was conducted among patients who attend HPT Clinics at selected hospitals in the Brong Ahafo region of Ghana [22]. A cross-sectional survey was deemed appropriate given that data would be collected at a single time point without any follow-up [23].

### 2.2. Study Setting

The Brong Ahafo region covers an area of 39,557 square kilometers in the middle-belt of Ghana. It was the second-largest region in the country (16.6% of land area) before its partition into three separate regions. The area shares boundaries with the Northern Region to the north, the Ashanti and Western Regions to the south, the Volta Region to the east, the Eastern Region to the southeast, and La Cote d'Ivoire to the west. The region occupies the forest and savannah zones of Ghana and is noted for cocoa, timber, grains, and tuber products [24].

### 2.3. Health Facilities

The region has a total of 690 health facilities: 30 hospitals, 82 health centers, 112 clinics, 43 private maternity homes, and 423 functional community health and preventive services (CHPS) facilities. There are 3,292 communities in the region; 2,800 are served by the GHS (85%) while the rest are served by CHAG and other NGOs [24].

### 2.4. Target Population

In this study, the target population was comprised of residents of the Brong Ahafo region who had been diagnosed as hypertensive or were taking prescription medication for HPT.

### 2.5. Inclusion and Exclusion Criteria

HPT patients currently on antihypertensive therapy and presenting for routine clinical visits at HPT clinics in the region were targeted for inclusion in the study if they had been diagnosed or were on medications for hypertension for at least 1 year, above 18 years of age, and voluntarily consented to be part of the study.

HPT patients who did not provide written consent to be part of the study or who were seriously ill were excluded from the study.

### 2.6. Sample Size

The sample size was calculated in StatCalc, EPI Info™ version 7.1.2.0 (Centers for Disease Control, Atlanta, USA). Epi Info™ is a data collection, management, analysis, visualization, and reporting software for public health professionals and a trademark of the Centers for Disease Control and Prevention (CDC). In a previous study, the prevalence of compliance to hypertension medication among a cross-section of patients selected from a hospital setting was approximately 42%. After correcting for population size, it was determined that 399 subjects constituted an adequate sample size to estimate medication adherence in this population with precision (*d* = 0.05) at a 95% confidence level.

### 2.7. Sampling Technique

Participants were recruited using a multistage sampling technique [25]. A list of publicly funded health facilities with organized HPT clinics in the region was obtained from the Regional Health Directorate in the first step. These facilities were used as the study sites. From the list (see Table 1), health facilities were randomly sampled using a computer-assisted randomization process. To ensure fair distribution of the total sample size to the respective health facilities' HPT clinics, a proportional quota approach was adopted. The average number of HPT clients enrolled in the various clinics was extrapolated from the out-patient attendance data of each health facility, which was obtained from the District Health Information Management System (DHIMS2), the official health data repository of the Ghana Health Service/Ministry of Health [24]. A systematic sampling technique was then employed to recruit participants at the facility level. The systematic sampling technique was done by selecting a random start near the beginning of the population list and then taking every unit equally spaced thereafter. The desired sample interval was obtained by dividing the population size by the desired sample size [26, 27].

### 2.8. Tool for Data Collection

The questionnaire was adapted from the work of Robinson [28], and it was also based on the constructs of the HBM. The final outlook of the questionnaire (Supplementary File S1) was informed by a review of pertinent literature from similar contexts [29, 30], a pilot study and expert advice, which situated the concepts being measured in the Ghanaian setting. Section A was made up of questions about sociodemographic variables such as gender, age, occupation, and education as well as medication history. These items were placed strategically at the beginning of interviews to cultivate rapport between the researcher and participant. In section B, “perceived susceptibility” to the adverse effects on HBP was evaluated in items 11 to 14, using a five-point scale in line with earlier research [28]. The “perceived severity” of the adverse effects on HBP was evaluated from items 15 to 19, measured on a five-point scale. “Perceived benefits” of following clinical counsel were evaluated from items 20 to 26 using a five-point scale. “Perceived barriers” to optimal medication behaviours were evaluated in items 27 to 31. “Cues to action,” which refers to sources of stimuli for initiating positive medication behaviour, were evaluated in items 32 to 35 on a scale indicating the frequency of encountering such stimuli. “Self-efficacy,” which deals with intrinsic motivation for pursuing recommended actions, was evaluated in items 36 to 41 on a scale of certainty. Medication adherence was measured as a cumulative response to a set of questions ascertaining whether participants had intentionally or unintentionally been able to take their prescribed medication as scheduled.

### 2.9. Reliability and Validity

A pilot study was done using the research instrument in the Wamfie District Hospital. The purpose of the pilot study was to test logistics and clinical scenarios and also plan for the broader field study, as well as to conduct a psychometric analysis of the questionnaire, testing for validity and reliability. The results of the pilot study were evaluated by the second and third authors who are experts in the field of quantitative research. The required changes were made to the questionnaire for data collection. The reliability and validity of the adapted scales have been established in various studies [5, 12, 31, 32]. To further ascertain the reliability following the adaptation, Cronbach's reliability coefficients ranging from 0.00 to 1.00, with higher coefficients indicating higher levels of reliability, were used to determine the validity and the reliability of the questionnaire and reported.

### 2.10. Statistical Analysis

Data from various study sites were captured using uniform Microsoft Excel worksheets (Supplementary File S2). Characteristics were presented as frequencies, percentages, means, medians, interquartile ranges, and standard deviations as appropriate. Sociodemographic characteristics and HBM predictors' association with noncompliance status were tested with a chi-square test. In the case of small subgroups, Fisher's exact test was used. Significant variables were considered for a binary logistic regression model. The multivariate binary logistic regression model was used to determine the odds of the predictors on the dependent variable (hypertension medication at 0 and 1 levels: “0” for compliance to hypertension medication and “1” for noncompliant to hypertension medication). A forward stepwise regression selection was adopted for the outcome and the predictor variables. Three models designated model 0, 1, and 2 were fitted. Model 0 presents the variance in the dependent variable without the predictor variables, while models 1 and 2 contain the factors and the constructs of the HBM, respectively. In model 1, parameters such as age and level of education were included, while in model 2, parameters such as susceptibility, severity, barriers, and cues to action were also included concurrently. The level of significance was set at *p* < 0.05, and all tests were two-sided. All statistical analyses were completed using the SPSS software (version 25; SPSS Inc, Chicago, IL).

### 2.11. Ethical Clearance

Ethics clearance for commencement of the study was obtained from the Ethics Review Committee of the Christian Health Association of Ghana (CHAG). Respondents were adequately informed of the purpose, nature, procedures, risks, and hazards of the study. Points emphasized included anonymity, confidentiality, and the freedom to decline to participate at any time without penalty. Permission was also sought from the respective health facilities where respondents were recruited. Written consent was obtained from all study participants.

## 3. Results

### 3.1. Demographic Characteristics of Respondents

The mean age of respondents was 52.7 (±10.4) years (range 27–78 years). Both males and females were represented in roughly equal proportions (females: 50.9%, *n* = 203; males: 49.1%, *n* = 196). Most of the respondents (79.7%, *n* = 318) were married; 10.0% (*n* = 40) were separated, while 9.0% (*n* = 36) were single. The majority of respondents reported having family care and support (90.5%, *n* = 360). Approximately a third of participants (32%, *n* = 128) had received tertiary education; 31.1% (*n* = 124) had received basic education; and 26.1% (*n* = 104) had completed secondary or vocational education. A tenth of participants indicated that they only had nonformal education (10.8%, *n* = 43). Regarding occupation, respondents were private workers (36.3%, *n* = 145), government workers (34.3%, *n* = 137), or subsistence farmers (23.1%, *n* = 92; Table 2).

### 3.2. Distribution of History of Hypertensive Medication

The median duration of the respondent's hypertensive diagnosis and medication history was five years. The majority (56.9%, *n* = 227) of respondents reported no comorbidities, while others reported a history of diabetes (33.3%, *n* = 133), renal failure (3.3%, *n* = 13), or heart failure (6.5%, *n* = 26). National Health Insurance Scheme (NHIS) coverage was higher (82.4%, *n* = 328) than private insurance coverage (11.1%, *n* = 44). Despite the high proportion of insurance cover, a small minority group (6.5%, *n* = 26) were without any form of insurance cover. A considerable proportion (74.2%, *n* = 296) of the participants were on antihypertensive medication, while 25.8% (*n* = 103) were on both antidiabetic and hypertensive medication (Table 3).

### 3.3. Prevalence of Nonadherence to Hypertensive Medication

**Figure 1:**
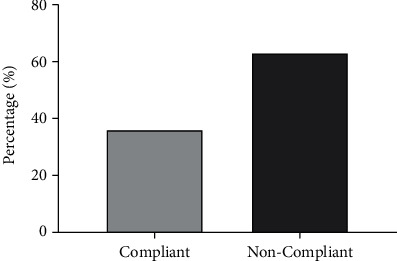
Prevalence of noncompliance with hypertension medication.

Figure 1 shows the prevalence of nonadherence among the study population. The prevalence of nonadherence to hypertensive medication stood at 63.7% (*n* = 254) with only a third (36.3%, *n* = 145) of the population reporting adherence to instructions for taking their medication.

### 3.4. Association between Sociodemographic Characteristics and Medication Nonadherence

The cross-sectional association between selected sociodemographic variables and medication nonadherence is shown in Table 4. In chi-square analysis, education status was significantly associated with nonadherence to hypertensive medication (*χ*^2^ (3, *N* = 399) = 4.179, *p*=0.041). Respondents who spent more years in formal education were less likely to forfeit their medication regimen (adjusted odds ratio (aOR) = 0.559, 95% CI: 0.360–0.867,*p*=0.009). An independent sample *t*-test indicated a significantly higher mean age for individuals classified as compliant (mean: 54.18, SD = 12.35) compared to those classified as noncompliant (mean: 51.84, SD = 9.06; *t* (397) = 1.993, *p*=0.047). Increasing age was associated with a decreased likelihood of exhibiting noncompliance (aOR = 0.972, 95% CI: 0.952–0.992, *p*=0.007).

### 3.5. Association between Constructs of the HBM and Nonadherence Behaviour

A binary logistic regression was performed to ascertain the effects of HBM constructs: perception of susceptibility, severity, barriers, cues to action, and benefit on the likelihood that participants will be classified as noncompliant. The model was a good fit (*χ*^2^ (8) = 14.372, *p*=0.073). The model explained 33.3% (Nagelkerke *R*^2^) of the variance in noncompliance and correctly classified 71.2% of cases. As shown in Table 5, respondents with a high perception of susceptibility were more likely to be classified as nonadherents to hypertension medication (aOR = 3.889, 95% CI: 2.254–6.710, *p* < 0.001). Having a low perception of the severity of hypertension was associated with increased odds of being classified as a nonadherent to hypertensive medication (aOR = 4.884, 95% CI: 2.753–8.663, *p* < 0.001). Having a high perception of barriers to action was associated with lower odds of nonadherence to hypertension medication (aOR = 0.275, 95% CI: 0.149–0.506, *p* < 0.001). Participants with a high perception of cues to action were more likely to be classified as adherent and less likely to be classified as nonadherents to hypertension medication (aOR = 0.363, 95% CI: 0.215–0.612, *p* < 0.001). Having a high perception of benefits did not have a significant effect on respondent noncompliance (aOR: 0.687, 95% CI: 0.353–1.337, *p*=0.269).

## 4. Discussion

In line with the growing interest and requirement for patient-centric approaches to disease management, a better appreciation of patient-level contextual factors remains a valuable resource for noncommunicable disease programmes and public health managers. The present study examined predictors of nonadherence to HPT treatment in the Brong-Ahafo Region of Ghana using the constructs of the Health Belief Model. Consistent with other studies in Ghana and other locales [12, 16, 32], this study revealed a high prevalence of nonadherence to medication among hypertensive patients. The prevalence of 63.7% (*n* = 254) nonadherence shows that nonadherence to pharmacological treatment is a common phenomenon among HPT patients in Ghana. There is an urgent need for the health promotion units in the Region to intensify their education programmes on the need for adherence to HPT medication to avert short- and long-term cardiovascular complications [33].

Fewer years spent in school contributed to the pattern of poor medication adherence observed. Respondents who had not been formally educated beyond the basic stage were more likely (*p* < 0.009) to forfeit their HPT medications (Table 4). Concordant results have been reported by other studies around the country [1, 12, 34]. Our data is also in line with a recent systematic review where a low level of education was significantly associated with low knowledge of the HPT condition and of the value of pharmacological treatment, which invariably has a negative consequence on medication adherence especially in rural communities where HPT cases are increasing [35].

In contrast to reports from other settings, patients with high perceived susceptibility and perceived severity in this study were more likely to be classified as noncompliant with HPT medication [31, 33]. In a previous investigation into the psychosocial experiences of HPT patients in the region, key subthemes that emerged from the data were suicidal ideations and resignation to fate [1]. Although they were well-informed about the risk posed by complications of HPT, as seen from the responses to statements such as “I am worried about becoming sick or disabled from high blood pressure” and “Having high blood pressure could lead to serious health problems for me,” rather than resulting in positive medication behaviour, participants were more deeply troubled by the psychosocial burden of an incurable disease and life-long therapy. These challenges give rise to unhealthy mental adaptations such as apathy to care, resentment, and resignation to fate as a result of the knowledge of HPT complications. Healthcare workers will need to be trained specifically on how to address this mentality to improve medication adherence. Another plausible explanation could be the side effects of HPT medications and the high cost of medications as a result of copayments demanded by health facilities from patients on the National Health Insurance Scheme (NHIS) [1]. Health professionals and policymakers should therefore work towards making medications for HPT patients available through the NHIS in order to reduce patient-level costs of HPT treatment in the hospitals to help improve adherence among HPT patients.

Patients classified as having a high perception of barriers to medication behaviour were more likely to exhibit positive medication-taking behaviour. This finding lies in contrast to previous studies in similar settings [12, 36], which reported that perceived barriers were strongly associated with noncompliance with HPT medication. Although HPT patients experience a great number of challenges that can impair medication behaviour, a number of adaptations to these challenges have also been reported. These adaptations or coping strategies can be classified under at least three subthemes: health system support, social support, and religiosity. It is quite possible that participants who felt supported by nurses, doctors, dietitians, family members, and religious systems would exhibit positive medication behaviour [1, 37, 38]. This was evident from the observation that study participants with a high perception of cues to action were more likely to be classified as adherent and less likely to be classified as nonadherents to hypertension medication. These findings suggest that poor medication behaviour could be improved by a better appreciation of patient-level factors and mechanisms by which psychosocial variables shape medication behaviour. Better collaboration between healthcare professionals and patients with HPT should lead to positive medication behaviour and ensure optimum health outcomes.

### 4.1. Strengths and Limitations of the Study

In terms of strengths, this study is one of the few studies that highlights the application of the HBM constructs in predicting factors contributing to nonadherence to HPT medication in Ghana. However, the study has some limitations. To begin with, the study was a cross-sectional survey and this limited our ability to offer some explanations concerning the causal correlation between the study variables and nonadherence. The respondents for the study were all from one region, thus the findings may not be representative of the entire country.

## 5. Conclusion

This current study has successfully expanded the utility of the HBM in understanding patient noncompliance to hypertensive medication by triangulating previous data on challenges faced by HPT patients. The study revealed a high prevalence of noncompliance to HPT medication linked to low exposure to higher education among HPT patients in Ghana. Additionally, healthcare workers and NCD programme managers could improve compliance to HPT medication by targeting patient-level factors represented as constructs of the HBM. Our results show that a high perception of susceptibility and severity does not necessarily translate into positive medication behaviour, whereas a better perception of barriers does not necessarily result in negative medication behaviour. However, strong cues to action remain useful in reducing medication noncompliance. There is therefore the need for the health promotion unit in the region to redesign health interventions that will help improve HPT adherence based on the constructs of the Health Belief Model.

## Figures and Tables

**Table 1:** Allocation of the sample to health facilities.

Name of facility	Estimated monthly HPT cases based on population prevalence of 28%				5-Year average of estimated HPT cases	Proportional allocation (%)	Allocated sample
	2014	2015	2016	2017			
Ahmadiyyah (Techiman) Mission Hospital	1,228	996	1,022	1,002	1,062	3	12
Atebubu Hospital	1,382	999	1,143	1,002	1,132	3	12
Bechem Government Hospital	1,301	1,475	1,667	1,329	1,443	4	16
Dormaa East District Hospital	869	976	838	715	849	2	9
Dormaa Presbyterian Hospital	1,846	1,566	1,810	1,936	1,790	5	20
Dormaa West District Hospital	412	418	493	554	470	1	5
Drobo St Mary Hospital	2,669	2,683	2,433	1,862	2,412	7	26
Duayaw Nkwanta St John of God Hospital	2,214	1,937	1,661	2,018	1,957	5	22
Goaso Municipal Hospital	1,485	1,224	1,245	1,186	1,285	4	14
Holy Family (Berekum) Hospital	2,817	1,725	2,620	1,763	2,231	6	25
Hwidiem St Elizabeth Hospital	2,818	2,832	2,323	2,094	2,517	7	28
Kintampo Muni Hospital	1,937	1,415	1,383	1,280	1,504	4	17
Kintampo South District Hospital	772	786	755	701	753	2	8
Mathias Hospital Yeji	1,974	2,160	1,931	1,244	1,827	5	20
Nsawkaw Hospital	932	964	1,027	959	970	3	11
Sampa Government Hospital	1,011	1,037	978	940	991	3	11
Sene District Hospital	751	766	924	905	836	2	9
St Theresa Hospital-Nkoranza	2,098	1,187	1,235	1,120	1,410	4	15
Sunyani Municipal Hospital	1,623	1,290	1,365	1,471	1,437	4	16
Sunyani Regional Hospital	3,488	3,104	2,494	2,050	2,784	8	31
Sunyani SDA Hospital	1,505	1,211	1,244	1,268	1,307	4	14
Techiman Holy Family Hospital	5,122	4,724	3,002	2,892	3,935	11	43
Wenchi Methodist Hospital	1,809	1,615	1,583	1,031	1,510	4	17
**Total**	**42,064**	**37,089**	**35,176**	**31,321**	**36,412**	**100**	**400**

Source: Extrapolated from District Health Information Management System (DHIMS2).

**Table 2:** Demographic characteristics of respondents.

Parameter	Frequency	Percentage (%)
*Total*	*399*	*100.0*
Age (mean ± SD)	52.69 ± 10.42	27–78
Gender		
Male	196	49.1
Female	203	50.9
Marital status		
Single	36	9.0
Married	318	79.7
Cohabitating	5	1.3
Separated/widowed	40	10.0
Family care and support		
Yes	360	90.5
No	38	9.5
Level of education		
Nonformal	43	10.8
Basic	124	31.1
Secondary/vocational	104	26.1
Tertiary	128	32.0
Occupation		
Unemployed	13	3.3
Government work	137	34.3
Private work	145	36.3
Farmer	92	23.1
Student	12	3.0

Data are presented as frequency and percentage.

**Table 3:** Clinical characteristics of study participants.

Parameter	Frequency	Percentage (%)
*Total*	*399*	*100*
Presence of other illnesses		
None	227	56.9
Diabetes	133	33.3
Renal failure	13	3.3
Heart failure	26	6.5
Type of health insurance		
None	26	6.5
NHIS	328	82.4
Private	44	11.1
Current medication		
Antihypertensive	296	74.2
Antidiabetic and hypertensive	103	25.8
**Years diagnosed with HBP (median, IQR)**	5	3–7
**Years on medication (median, IQR)**	5	3–8

Data are presented as frequency and percentage. IQR: interquartile range and HBP: high blood pressure.

**Table 4:** Association between sociodemographic characteristics and respondents' medication nonadherence.

Parameter	Noncompliance status			*χ * ^2^-value (*p*-value)/*t*-statistic (*p*-value)	aOR (95% CI)	*p*-value
	Compliant	Noncompliant	Total			
Total	145 (36.3%)	254 (63.7%)	399 (100.0%)			
Age (mean ± SD)	54.18 ± 12.35	51.84 ± 9.06	52.69 ± 10.42	1.993 (0.047)	0.972 (0.952–0.992)	0.007^*∗*^
Gender				0.333 (0.603)		
Male	74 (51.0%)	122 (48.0%)	196 (49.1%)			
Female	71 (49.0%)	132 (52.0%)	203 (50.9%)			
Level of education				4.179 (0.041)		
Up to basic level	51 (30.5%)	116 (69.5%)	167 (41.9%)		1	
Secondary/tertiary	94 (40.5%)	138 (59.5%)	232 (58.1%)		0.559 (0.360–0.867)	0.009^*∗*^
Any other illness						
None	74 (51.0%)	153 (60.2%)	227 (56.9%)	3.874 (0.275)		
Diabetes	57 (39.3%)	76 (29.9%)	133 (33.3%)			
Renal failure	5 (3.4%)	8 (3.1%)	13 (3.3%)			
Heart failure	9 (6.2%)	17 (6.7%)	26 (6.5%)			
Type of health insurance cover						
None	5 (3.5%)	21 (8.3%)	26 (6.5%)	3.496 (0.174)		
NHIS	122 (84.7%)	206 (81.1%)	328 (82.4%)			
Private	17 (11.8%)	27 (10.6%)	44 (11.1%)			
Medication currently taken						
Antihypertensive medication	104 (71.7%)	192 (75.6%)	296 (74.2%)	0.721 (0.407)		
Antidiabetic and hypertensive medication	41 (28.3%)	62 (24.4%)	103 (25.8%)			

Data are presented as frequency and percentage in parenthesis, *f* (%). SD: standard deviation, NHIS: National Health Insurance Scheme, aOR: adjusted odds ratio, *χ*^2^-value: chi-square value, and CI: confidence interval. *p*-Value < 0.05 is considered significant.

**Table 5:** Association of constructs of HBM with respondent's medication nonadherence.

Parameter	Noncompliance status			*χ * ^2^-value (*p*-value)	aOR (95% CI)	*p*-value
	Compliant	Noncompliant	Total			
*Total*	***145 (36.3%)***	***254 (63.7%)***	***399 (100.0%)***			
Susceptibility				**38.886 (0.001)**		
Low	103 (71.0%)	98 (38.6%)	201 (50.4%)		Reference	
High	42 (29.0%)	156 (61.4%)	198 (49.6%)		3.889 (2.254–6.710)	**0.001 ** ^*∗*^
Severity				**50.552 (0.001)**		
Low	117 (80.7%)	112 (44.1%)	229 (57.4%)		Reference	
High	28 (19.3%)	142 (55.9%)	170 (42.6%)		4.884 (2.753–8.663)	0.**001**^*∗*^
Barriers				**8.982 (0.003)**		
Low	66 (45.5%)	155 (61.0%)	221 (55.4%)		Reference	
High	79 (54.5%)	99 (39.0%)	178 (44.6%)		0.275 (0.149–0.506)	0.**001**^*∗*^
Cues to action				**13.971 (0.001)**		
Low	54 (37.2%)	144 (56.7%)	198 (49.6%)		Reference	
High	91 (62.8%)	110 (43.3%)	201 (50.4%)		0.363 (0.215–0.612)	0.**001**^*∗*^
Self-efficacy				**3.345 (0.069)**		
Low	46 (31.7%)	104 (40.9%)	150 (37.6%)			
High	99 (68.3%)	150 (59.1%)	249 (62.4%)			
Benefit				**9.539 (0.003)**		
Low	112 (77.2%)	158 (62.2%)	270 (67.7%)		Reference	
High	33 (22.8%)	96 (37.8%)	129 (32.3%)		0.687 (0.353–1.337)	0.269

Data are presented as frequency and percentage in parenthesis, *f* (%). SD: standard deviation, NHIS: National Health Insurance Scheme, aOR: adjusted odds ratio, *χ*^2^-value: Chi-square value, and CI: confidence interval. ^*∗*^*p*-value <0.05 is considered statistically significant.

## Data Availability

The relevant data for this study are included within the article and its supplementary information files (Supplementary Files: S1 and S2).

## Supplementary Materials

Supplementary Materials Supplementary File: S1: Questionnaire. Supplementary File S2: Data file.
