# Supplementary material for: Predictors of Nonadherence to Medications among Hypertensive Patients in Ghana: An Application of the Health Belief Model
Source: Int J Hypertens. 2022 Aug 24;2022:1418149. doi: 10.1155/2022/1418149 (PMC9433278; doi:10.1155/2022/1418149)
Supplement: Supplementary Materials — Supplementary File: S1: Questionnaire. Supplementary File S2: Data file. [file 1418149.f1.zip › S1;Questionnaire.docx]

# Appendix 1

**Adherence to Hypertension Study in the Brong-Ahafo Region Ghana**

You are among adults in Brong-Ahafo Region who have agreed to this Study. Hopefully, your participation will help improve the health of people living with High Blood Pressure in the Brong Ahafo Region. Please take the next 20 minutes to answer the following questions. Your participation is greatly appreciated.

**SECTION A**

| 1. Indicate age: |  | | |
| --- | --- | --- | --- |
| 1. Indicate gender: | [M] |  | [F] |
| 1. Indicate marital status | [ ] Single  [ ] Married  [ ] Cohabiting  [ ] Separated/widowed | | |
| 1. Indicate presence of family support | [YES] [NO] | | |
| 1. Highest level of education **attained**:   6.What is your occupation | [ ] Non-formal  [ ] Basic  [ ] Secondary/Vocational  [ ] Tertiary | | |
| 1. How long ago were you diagnosed with High Blood Pressure? |  | | |
| 1. Has the doctor told you you have any other illness? *Indicate diagnosis of other complication of hypertension if any.* |  | | |
| 1. Indicate type of health insurance cover if any | [ ] None  [ ] NHIS  [ ] Private | | |
| 1. What medication are you taking currently? *Verify all medication in the past 6months from folder and state here*. |  | | |
| 1. How long have you been taking medication for blood pressure management? |  | | |

**SECTION B**

| **Kindly indicate your best response to the following statements [susceptibility4_severity5]** | **Strongly**  **Agree** | **Agree** | **Uncertain** | **Disagree** | **Strongly Disagree** |
| --- | --- | --- | --- | --- | --- |
| 1. I am worried about becoming sick or disabled from High Blood Pressure. |  |  |  |  |  |
| 1. At my age I ***probably will not*** become very sick or disabled from High Blood Pressure |  |  |  |  |  |
| 1. There is a chance that I will become very sick or disabled from High Blood Pressure |  |  |  |  |  |
| 1. I am too healthy to become very sick or disabled from High Blood Pressure. |  |  |  |  |  |
| 1. Having High Blood Pressure could lead to serious health problems for me |  |  |  |  |  |
| 1. Having High Blood Pressure could lead to serious physical problems for me |  |  |  |  |  |
| 1. Having High Blood Pressure could lead to financial problems for me |  |  |  |  |  |
| 1. Having High Blood Pressure could cause me to have a stroke. |  |  |  |  |  |
| 1. Having High Blood Pressure could cause me to have a heart attack |  |  |  |  |  |
| *Cutting down on salt, losing weight, increasing exercise, eating fruits and vegetables, abstaining from or drinking alcohol in moderation, and taking prescribed medication can affect blood pressure.*  **Circle your response to the following statements [benefits7_barriers5]** | **Strongly**  **Agree** | **Agree** | **Uncertain** | **Disagree** | **Strongly Disagree** |
| 1. Doing these things can help me stay healthy |  |  |  |  |  |
| 1. Doing these things can help me live longer. |  |  |  |  |  |
| 1. Doing these things can reduce my chances of having a stroke |  |  |  |  |  |
| 1. Doing these things can reduce my chances of having a heart attack |  |  |  |  |  |
| 1. Doing these things can reduce my chances of having early death. |  |  |  |  |  |
| 1. Doing these things can reduce my chances of becoming disabled. |  |  |  |  |  |
| 1. Doing these things gives me a sense of accomplishment |  |  |  |  |  |
| 1. Doing these things takes too much money |  |  |  |  |  |
| 1. Doing these things is too difficult to understand |  |  |  |  |  |
| 1. Doing these things is hard to stick to when I am with family and friends |  |  |  |  |  |
| 1. Doing these things does not seem to make a difference in how I feel |  |  |  |  |  |
| 1. Doing these things makes me feel worse than if I do nothing at all |  |  |  |  |  |
| **Kindly indicate your response to the following statements [cues4]** | **Never** | **Rarely** | **Occasionally** | **Sometimes** | **Frequently** |
| 1. I get advice on how to lower my blood pressure from my doctor, nurse, or health care provider |  |  |  |  |  |
| 1. I get advice on how to lower my blood pressure from friends or family members. |  |  |  |  |  |
| 1. I pay attention to media (T.V., radio, bill board signs) messages about how to lower my blood pressure |  |  |  |  |  |
| 1. I read written materials (pamphlets, brochures, fact sheets, or postcards) with messages about how to lower my blood pressure. |  |  |  |  |  |
| **What is the chance that you *could take* the following actions to control your blood pressure? [self efficacy6]**  **Kindly indicate your best response** | **No Chance**  **at All** | **A Slight Chance** | **A 50/50 Chance** | **A Good Chance** | **Completely**  **Certain** |
| 1. Cut down on salt |  |  |  |  |  |
| 1. Lose weight |  |  |  |  |  |
| 1. Increase your amount of exercise |  |  |  |  |  |
| 1. Eat a diet high in fruits and vegetables |  |  |  |  |  |
| 1. Abstain from or drink alcohol in moderation |  |  |  |  |  |
| 1. Take a prescribed medication |  |  |  |  |  |

**SECTION C**

| **Kindly indicate your best response to the following statements** | **YES** | |  | **NO** | |
| --- | --- | --- | --- | --- | --- |
| 1. Do you occasionally forget to take your hypertension medications? |  | |  |  | |
| 1. For the past 2 weeks, were there any days that you did not take your hypertension medicine? |  | |  |  | |
| 1. Have you ever reduced or stopped taking your medication because you felt worse when you take the drugs without informing your Doctor? |  | |  |  | |
| 1. Do you sometimes forget to go along with your medications when you embark on a journey? |  | |  |  | |
| 1. Did you take your high blood pressure medicine yesterday? |  | |  |  | |
| 1. When you feel that your blood pressure is under control, do you sometimes stop taking your medications? |  | |  |  | |
| 1. The intake of medication every day is really inconveniencing for some people. Do you at times feel troubled about sticking to your blood pressure treatment regimen? |  | |  |  | |
|  | **Never** | **Rarely** | **Occasionally** | **Often** | **Always** |
| 1. How often do you have difficulty remembering to take all your blood pressure medication? |  |  |  |  |  |
| 1. Do you follow your doctor’s instructions for taking blood pressure medication? |  |  |  |  |  |
